# Supplementary material for: Evaluation of search-enabled pretrained Large Language Models on retrieval tasks for the PubChem database
Source: Bioinform Adv. 2025 Mar 24;5(1):vbaf064. doi: 10.1093/bioadv/vbaf064 (PMC12073969; doi:10.1093/bioadv/vbaf064)
Supplement: vbaf064_Supplementary_Data [file vbaf064_supplementary_data.zip › Supporing_File_1___PubChem_LLM_Retrieval.pdf]

Evaluation of search-enabled Pre-trained Large Language Models on retrieval tasks for the PubChem Database

Ash Sze and Soha Hassoun
Tufts University

November 21, 2024

Supporting File 1

Detailed Results on the Protocol

We describe the details of evaluating the remainder of the protocols in details, highlighting values and limitations that are specific to each case.

Protocol 5. The fifth protocol aims to find drugs that target the human type-1 angiotensin II receptor gene. The data originates from several sources within PubChem, including DrugBank. The PubChem web interface guides users to search for "type 1 angiotensin II receptor," select the "Genes" tab and click the entry with "Human," navigate to the 7.1 "Chemical-Gene Interactions" subsection, sort by "Data Source" to view all interacting compounds from DrugBank, and download the necessary sections (see full results in the appendix).

GPT-4o provides a list of drugs that interact with the gene encoding the human type-1 angiotensin II receptor. The response is correct but incomplete. GPT-4o lists the drugs Valsartan, Olmesartan, Telmisartan, Irbesartan, Candesartan, and Azilsartan medoxomil, which is only 7 out of the 14 total drug interactions from DrugBank. Attempting to enhance the prompt by asking for a complete list, or all of the data lead to faulty code generation or an output unchanged from the initial output.

There is no programmatic way to directly access the gene-chemical interactions table. There is no silver response (refer to figure S1 for a full protocol summary).

Protocol 6. The sixth protocol aims to get the bioactivity data of all chemicals tested against the human type-1 angiotensin II receptor (AT1R) and its rat ortholog. The data originates from various sources within PubChem. The PubChem web interface guides users to access the protein summary page for "type 1 angiotensin II receptor," navigate to the "tested compounds" section, download the list of tested compounds and their bioactivity data, and repeat the process for "P29089 (Norway rat)" under the orthologous protein section (see full results in the appendix).

GPT-4o provides a summary of the bioactivity data for the most important chemicals tested against the human type-1 angiotensin II receptor and its rat ortholog. GPT-4o cannot directly search PubChem and thus only provides possible bioactivity types, a general overview of the AT1R receptor, and listings of human AT1R interactions for compounds losartan, Olmesartan, and Candesartan, and rat AT1R interactions for compounds Valsartan and Irbesartan. GPT-4o does not explain how it shortlisted these compounds, and descriptions of the compounds are generic and do not reflect the gold answer. Enhanced prompting by re-querying and asking for exact lists of compounds did not improve the quality of the responses for this protocol.

To retrieve similar information programmatically, we utilized PUG to perform a concise bioactivities search using the gene IDs 185 and 81638, corresponding to AGTR1 and AGTR1b respectively. This method involved finding the relevant gene IDs and using URLs to obtain detailed bioactivity data.

We also prompted GPT-4o with a programmatic access approach to provide PUG links for concise gene searches on gene ID 185 and gene ID 81638. The prompt included specific criteria to return a summary of bioactivity data for these gene IDs. GPT-4o generated incorrect PUG URLs due to an invalid input domain (refer to figure S2 for a full protocol summary).

Protocol 7. The seventh protocol aims to find compounds annotated with specific classifications or ontological terms using PubChem. The data originates from PubChem's chemical database. The PubChem web interface guides users to the Classification Browser, selecting "MeSH" for classification and "Compound" for data, searching for "Antihypertensive agents" and saving as "1," repeating for "anti-arrhythmia agents" and saving as "2," and using the AND operator to search for results matching both classifications (see full results in the appendix).

GPT-4o cannot perform a direct search on PubChem and is thus unable to use the Boolean search function required for this protocol. The original protocol requires the user to create two separate searches and use the Boolean operator AND to find results that match both statements. While PubChem has 68 results for this search, none of them correspond to GPT-4o's answers. GPT-4o instead generates hypothetical datasets of chemicals and interactions, which do not accurately reflect the actual data available in PubChem. Enhanced prompting did not improve the quality of the responses for this protocol.

There is currently no way to programmatically access classifications or search by MeSH annotations via PUG-REST. The PUG-REST tutorial acknowledges this as a feature that may be added in the future based on demand. Thus, there is no silver answer for this protocol through programmatic access (refer to figure S3 for a full protocol summary).

Protocol 8. The eighth protocol aims to find stereoisomers and isotopomers of a compound using an identity search in PubChem. The data originates from PubChem's comprehensive chemical database. The PubChem web interface guides users to search for "CID 60846 structure," select "Identity" and select "Same Isotope" in "Settings" for stereoisomers and "Same stereo" for stereoisotopomers and download the results as a CSV file (see full results in the appendix).

GPT-4o provides a list of stereoisomers and isotopomers for Valsartan (CID 60846). The response includes the (S)-enantiomer as a commonly known stereoisomer, and Valsartan-d3 and [3H]Valsartan as isotopomers. However, the names and formatting of these chemical compounds differ from PubChem's data, indicating discrepancies. The response does not match the gold answer from PubChem. Attempting to enhance the prompt by changing "find" to "list" or "retrieve" led to faulty code generation. Re-querying for the correct answer leads to a change in output compounds that was still incorrect.

To retrieve similar information programmatically, we used PUG to perform a fast identity structure search for stereoisomers and isotopomers using the CID 60846. This method involved setting "identity_type" as "same_stereo" for stereoisotopomers and "same_isotope" for stereoisomers and using specific URLs to obtain the silver answer which matched the gold answer.

We prompted GPT-4o with a programmatic access approach to provide PUG links for a fast identity search using the same stereo and same isotope settings for CID 60846. However, the output generated by GPT-4o was incorrect, resulting in PUG-REST faults due to invalid operations. (refer to figure S4 for a full protocol summary)

Reference Protocol

1. Visit the PubChem homepage
2. Search for "type 1 angiotensin II receptor"
3. Select the "Genes" tab and click the entry with "Human"
4. Go to the 7.1 "Chemical-Gene Interactions" subsection
5. Sort by "Data Source" to view all interacting compounds from DrugBank and download sections as needed.

Programmatic Access Protocol

There is currently no method to programmatically retrieve the "Chemical-Gene Interactions" table data directly via PUG. There is no silver answer for this protocol.

GPT Generation via Search

GPT Prompt: "Based on information only from PubChem, find the most important drugs that interact with the gene encoding the human type-1 angiotensin II receptor, which is the target of losartan."

GPT output: List of drugs including Valsartan, Olmesartan, Telmisartan, Irbesartan, Candesartan, Eprosartan, and Azilsartan medoxomil.

GPT Generation via Programmatic Prompt

GPT Programmatic Prompt and **PUG-based GPT output** is not applicable because there is no programmatic access method or silver answer.

Figure 1: Protocol #5 for finding drugs that target a particular gene, made specific for the gene type 1 angiotensin receptor (human).

Reference Protocol

1. Access the protein summary page for "type 1 angiotensin II receptor" similar to protocol 5
2. Go to the "tested compounds" section
3. Download the list of tested compounds and their bioactivity data
4. Repeat the process for "P29089(Norway rat)" under the orthologous protein section.

Programmatic Access Protocol

There is a programmatic way to access the bioactivity data of chemicals tested against a protein. The silver answer is more detailed than the table found through PubChem

1. Find the gene id for AGTR1 and AGTR1b (185 and 81638)
2. Perform a concise bioactivities search using the gene IDs

https://pubchem.ncbi.nlm.nih.gov/rest/pug/gene/geneid/185/concise/JSON
https://pubchem.ncbi.nlm.nih.gov/rest/pug/gene/geneid/81638/concise/JSON

GPT Generation via Search

GPT Prompt: "Based on information only from PubChem, create or link to a summary for and list the bioactivity data of the most important chemicals tested against the human type-1 angiotensin II receptor and its rat ortholog."

GPT output: List of summarized bioactivity data for human AT1R including losartan, Olmesartan, and Cadesartan, and for rat AT1R including Valsartan, and Irbesartan.

GPT Generation via Programmatic Prompt

GPT Programmatic Prompt: "Provide the PUG URL for a concise gene search on gene ID 185 and another URL for gene ID 81638"

Pug-based GPT Output:
https://pubchem.ncbi.nlm.nih.gov/rest/pug/genes/geneid/185/summary/JSON (PUG-REST fault due to invalid input domain)
https://pubchem.ncbi.nlm.nih.gov/rest/pug/genes/geneid/81638/summary/JSON (PUGREST fault due to invalid input domain)

Figure 2: Protocol #6 for getting bioactivity data of all chemicals tested against a protein, made specific for the protein type 1 angiotensin II receptor (human and Norway rat).

Reference Protocol

1. Visit the PubChem Classification Browser
2. Select “MeSH” for classification and “Compound” for data
3. Search for “Antihypertensive agents” and save as “1”
4. Repeat for “anti-arrhythmia agents” and save as “2”
5. Use the AND operator to search ”1” and ”2” then view results

Programmatic Access Protocol

There is no way to programmatically access classifications or search by MeSH annotations via PUG-REST yet. The PUG-REST tutorial acknowledges this as a feature that may or more not be added in the future based on demand. There is no silver answer for this protocol.

GPT Generation via Search

GPT Prompt: “Based only on information from PubChem, list the chemicals with the same therapeutic uses as losartan, based on the MeSH annotations“

GPT output: Unable to directly perform the Boolean operations required to access the information, GPT is limited to creating hypothetical datasets for chemicals and interactions.

GPT Generation via Programmatic Prompt

GPT Programmatic Prompt and **PUG-based GPT output** is not applicable because there is no programmatic access method or silver answer.

Figure 3: Protocol #7 for finding compounds annotated with classifications or ontological terms, made specific for “antihypertensive agents” and “anti-arrhythmia agents.”

Reference Protocol

1. Visit the PubChem homepage
2. Search for “CID 60846 structure”
3. Click ”Identity” and select “Same Isotope” in “Settings” for stereoisomers and “Same stereo” for isotopomers
4. Download for a full CSV

Programmatic Access Protocol

There is a way to directly access the stereoisomers and isotopomers of a given CID. The silver answer is the gold answer.

1. Find the CID of the search compound (60846)
2. Perform a fast identity structure search using PUG, setting “identity_type” as “same_stereo” for stereo-isotopomers and “same_isotope” for stereoisomers.

https://pubchem.ncbi.nlm.nih.gov/rest/pug/compound/fastidentity/cid/60846/cids/TXT?identity_type=same_stereo
https://pubchem.ncbi.nlm.nih.gov/rest/pug/compound/fastidentity/cid/60846/cids/TXT?identity_type=same_isotope

GPT Generation via Search

GPT Prompt: “Based only on information from PubChem, find stereoisomers and isotopomers of a given compound, with valsartan (CID 60846)”

GPT output: Listed (S)-enantiomer as a commonly known stereoisomer, and Valsartan-d3 and [^{3}H]valsartan as isotopomers.

GPT Generation via Programmatic Prompt

GPT Programmatic Prompt: “Provide the PUG URLs for a fast identity search for the same stereo and the same isotope on CID 60846”

GPT Output:
https://pubchem.ncbi.nlm.nih.gov/rest/pug/compound/fastidentity/cid/60846/same_stereo/JSON (PUG-REST fault due to Invalid Operation)
https://pubchem.ncbi.nlm.nih.gov/rest/pug/compound/fastidentity/cid/60846/same_isotope/JSON (PUG-REST fault due to Invalid Operation)

Figure 4: Protocol #8 for getting stereoisomers and isotopomers of a compound through identity search, made specific for a search on “CID 60846 structure.”
